# Supplementary material for: Magnetic Nanoclusters Coated with Albumin, Casein, and Gelatin: Size Tuning, Relaxivity, Stability, Protein Corona, and Application in Nuclear Magnetic Resonance Immunoassay
Source: Nanomaterials (Basel). 2019 Sep 19;9(9):1345. doi: 10.3390/nano9091345 (PMC6781099; doi:10.3390/nano9091345)
Supplement: Supplementary file 1 [file nanomaterials-09-01345-s001.pdf]

Article

Magnetic Nanoclusters Coated with Albumin, Casein, and Gelatin: Size Tuning, Relaxivity, Stability, Protein Corona, and Application in Nuclear Magnetic Resonance Immunoassay

Pavel Khramtsov [1,2,*], Irina Barkina [2], Maria Kropaneva [1], Maria Bochkova [1], Valeria Timganova [1], Anton Nechaev [3], Il'ya Byzov [1,4], Svetlana Zamorina [1,2], Anatoly Yermakov [1,4,5] and Mikhail Rayev [1,2]

1 Laboratory of Ecological Immunology, Institute of Ecology and Genetics of Microorganisms of the Ural Branch of the Russian Academy of Sciences, branch of PSRC UB RAS, 13 Golev str., 614081 Perm, Russia; kropanevamasha@gmail.com (M.K.); krasnykh-m@mail.ru (M.B.); timganovavp@gmail.com (V.T.); ivbyzov@gmail.com (I.B.); mantissa7@mail.ru (S.Z.); yermakov@imp.uran.ru (A.Y.); mraev@iegm.ru (M.R.)

2 Department of Microbiology and Immunology, Biology Faculty, Perm State National Research University, 15 Bukirev str., 614000 Perm, Russia; i_barkina@mail.ru (I.B.)

3 Institute of Technical Chemistry of Ural Branch of the RAS, 3 Academician Korolev str., 614013 Perm, Russia; toxambj@gmail.com (A.N.)

4 M.N. Mikheev Institute of Metal Physics of the Ural Branch of the Russian Academy of Sciences, 18 S. Kovalevskoy str., 620108 Yekaterinburg, Russia

5 Ural Federal University named after the first President of Russia B.N. Yeltsin, 19 Mira str., 620002 Yekaterinburg, Russia

* Correspondence: khramtsovpavel@yandex.ru (P.K.); Tel.: +7-342-280-77-94

Received: 26 August 2019; Accepted: 18 September 2019; Published: date

Supporting Information

Experimental section

Synthesis of "small" (100-110 nm) BSA-coated Fe@C-NH$_2$ nanoparticles conjugated with streptavidin

325 μL of 20% BSA in H_2O was mixed with 975 μL of the 0.1M acetic buffer with pH 4, then 1300 μL of 10 mg/ml Fe@C-NH$_2$ suspension was added dropwise under vortex stirring. pH was adjusted to 7.25 with 1M NaOH. Resulting suspension (2500 μL) was added dropwise under vortex stirring to an equal volume of 25% glutaraldehyde solution (see *Materials* section) and incubated on rotating mixer (from now on rotation angle was set at 99 degrees, the rotation rate was 5 rpm) at room temperature for 30 min. Then, 5 ml of nanoparticles activated with glutaraldehyde were passed through the chromatography column (column XK 26/40, medium: Sepharose CL-6B, bed volume: 160 ml, elution speed: 0.65 ml/min, eluent: PBS) to remove the excess of BSA and glutaraldehyde. Fractions containing Fe@C-NH$_2$/BSA nanoparticles were collected and concentrated to approximately 2.5 ml. For concentrations, the suspension was placed in dialysis tubing (10 kDa MWCO, 2 ml per cm) and covered with the layer of 35 kDa PEG. In 2-3 hours concentrated suspension was removed from dialysis tubing and centrifuged at 1600g for 5 min. The supernatant contained Fe@C-NH$_2$/BSA nanoparticles activated with glutaraldehyde was divided into five equal portions (volume of the portion was approximately 500 μL). Each portion was added to 10 mg/ml streptavidin solution in PBS under vortex stirring; volumes were equalized with PBS. Final streptavidin to Fe@C-NH$_2$/BSA ratios were 10, 20, 40, 80 and 160 μg of streptavidin per 1 mg of nanoparticles. Conjugation of streptavidin was carried out overnight on rotating mixer at +4°C. Unreacted streptavidin was removed by gel-chromatography (column C 10/20, medium: Sepharose CL-6B, bed volume: 10 ml, elution speed: 0.07 ml/min, eluent: PBS). Fractions with the highest concentration of Fe@C-NH$_2$/BSA/Str nanoparticles were combined.

Synthesis of "medium" (170-180 nm) BSA-coated Fe@C-NH$_2$ nanoparticles conjugated with streptavidin

325 μL of 20% BSA in H_2O was mixed with 975 μL of 0.1M phosphate buffer pH 8, then 1300 μL of 10 mg/ml Fe@C-NH_2 suspension was added dropwise under vortex stirring. pH was adjusted to 7.25 with 1M HCl. Resulting suspension (2500 μL) was added dropwise under vortex stirring to an equal volume of 25% glutaraldehyde solution (see *Materials* section) and incubated on rotating mixer at room temperature for 30 min. Further procedures were as described for "small" Fe@C-NH_2/BSA/Str (see above).

Synthesis of "large" (210-220 nm) BSA-coated Fe@C-NH_2 nanoparticles conjugated with streptavidin

325 μL of 20% BSA in H_2O was mixed with 975 μL of 0.1M phosphate buffer pH 8, then ionic strength of mixture was adjusted to 0.5M with NaCl powder. After that, 1300 μL of 10 mg/ml Fe@C-NH_2 suspension was added dropwise under vortex stirring. pH was adjusted to 7.25 with 1M HCl. Resulting suspension (2500 μL) was added dropwise under vortex stirring to an equal volume of 25% glutaraldehyde solution (see *Materials* section) and incubated on rotating mixer at room temperature for 30 min. Further procedures were as described for "small" Fe@C-NH_2/BSA/Str (see above).

Synthesis of "small" (110-120 nm) casein-coated Fe@C-NH_2 nanoparticles conjugated with streptavidin

738 μL of 8.8% casein in H_2O was mixed with 562 μL of 0.1M acetic buffer pH 5, then 1300 μL of 10 mg/ml Fe@C-NH_2 suspension was added dropwise under vortex stirring. pH was adjusted to 7.25 with 1M HCl. Resulting suspension (2500 μL) was sonicated for 10 sec (30% amplification, 3 mm probe), added dropwise under vortex stirring to an equal volume of 25% glutaraldehyde solution (see *Materials* section) and incubated on rotating mixer at room temperature for 30 min. Further procedures were as described for "small" Fe@C-NH_2/BSA/Str (see above).

Synthesis of "medium" (190-210 nm) casein-coated Fe@C-NH_2 nanoparticles conjugated with streptavidin

738 μL of 8.8% casein in H_2O was mixed with 562 μL of 0.1M acetic buffer pH 5, then ionic strength of mixture was adjusted to 0.5M with NaCl powder. After that, 1300 μL of 10 mg/ml Fe@C-NH_2 suspension was added dropwise under vortex stirring. pH was adjusted to 7.25 with 1M HCl. Resulting suspension (2500 μL) was added dropwise under vortex stirring to an equal volume of 25% glutaraldehyde solution (see *Materials* section) and incubated on rotating mixer at room temperature for 30 min. Further procedures were as described for "small" Fe@C-NH_2/BSA/Str (see above).

Synthesis of "large" (240-260 nm) casein-coated Fe@C-NH_2 nanoparticles conjugated with streptavidin

738 μL of 8.8% casein in H_2O was mixed with 562 μL of 0.1M acetic buffer pH 5, then ionic strength of mixture was adjusted to 1M with NaCl powder. After that, 1300 μL of 10 mg/ml Fe@C-NH_2 suspension was added dropwise under vortex stirring. pH was adjusted to 7.25 with 1M HCl. Resulting suspension (2500 μL) was sonicated for 10 sec (30% amplification, 3 mm probe), added dropwise under vortex stirring to an equal volume of 25% glutaraldehyde solution (see *Materials* section) and incubated on rotating mixer at room temperature for 30 min. Further procedures were as described for "small" Fe@C-NH_2/BSA/Str (see above).

Synthesis of "small" (140-160 nm) gelatin B-coated Fe@C-NH_2 nanoparticles conjugated with streptavidin

650 μL of 10% gelatin B in H_2O was mixed with 650 μL of 0.1M acetic buffer pH 4, then 1300 μL of 10 mg/ml Fe@C-NH_2 suspension was added dropwise under vortex stirring. pH was adjusted to 7.25 with 1M NaOH. Resulting suspension (2500 μL) was sonicated on a water bath for 5 min (30% amplification, 3 mm probe), added dropwise under vortex stirring to an equal volume of 25% glutaraldehyde solution (see *Materials* section) and incubated on rotating mixer at +37°C for 30 min. All solutions were pre-warmed and kept at +37-40°C to prevent gelation. Further procedures were performed at room temperature as described for "small" Fe@C-NH_2/BSA/Str (see above).

Synthesis of "medium" (210-240 nm) gelatin B-coated Fe@C-NH_2 nanoparticles conjugated with streptavidin

650 μL of 10% gelatin B in H_2O was mixed with 650 μL of 0.1M phosphate buffer pH 8, then ionic strength of mixture was adjusted to 1M with NaCl powder. After that, 1300 μL of 10 mg/ml Fe@C-NH_2 suspension was added dropwise under vortex stirring. Resulting suspension (2500 μL) was sonicated on a water bath for 2 sec (30% amplification, 3 mm probe), added dropwise under vortex stirring to an equal volume of 25% glutaraldehyde solution (see *Materials* section) and incubated on rotating mixer at +37°C for 30 min. All solutions were pre-warmed and kept at +37-40°C to prevent gelation. Further procedures were performed at room temperature as described for "small" Fe@C-NH_2/BSA/Str (see above).

Synthesis of "large" (300-320 nm) gelatin B-coated Fe@C-NH_2 nanoparticles conjugated with streptavidin

1300 μL of 10 mg/ml Fe@C-NH_2 suspension was mixed with 650 μL of 0.1M phosphate buffer pH 8, then 650 μL of 10% gelatin B in H_2O was added dropwise under vortex stirring. Resulting suspension (2500 μL) was sonicated on water bath for 5 sec (30% amplification, 3 mm probe), centrifuged at 1600g for 5 min to remove large agglomerates, then supernatant was sonicated for another 5 sec and added dropwise under vortex stirring to equal volume of 25% glutaraldehyde solution (see *Materials* section) and incubated on rotating mixer at +37°C for 30 min. All solutions were pre-warmed and kept at +37-40°C to prevent gelation. Further procedures were performed at room temperature as described for "small" Fe@C-NH_2/BSA/Str (see above).

Synthesis of BSA-coated Fe@C-NH_2 functionalized with Streptococcal protein G (Fe@C-NH_2/BSA/G)

2250 μL of 10 mg/ml Fe@C-NH_2 suspension was added dropwise under vortex stirring to 625 μL of 20% BSA in H_2O, then 1595 μL of PBS and 30 μL of 1M NaOH were added to adjust pH to 7.2-7.6. Resulting suspension (4300 μL) was sonicated for 10 s and added dropwise under vortex stirring to an equal volume of 25% glutaraldehyde solution and incubated on rotating mixer at room temperature for 30 min. Then, 8.6 ml of nanoparticles activated with glutaraldehyde were passed through the chromatography column (column XK 26/40, medium: Sepharose CL-6B, bed volume: 160 ml, elution speed: 0.65 ml/min, eluent: PBS) to remove the excess of BSA and glutaraldehyde. Fractions containing Fe@C-NH_2/BSA nanoparticles were collected and concentrated to approximately 3-4 ml. For concentrations, the suspension was placed in dialysis tubing (10 kDa MWCO, 2 ml per cm) and covered with the layer of 35 kDa PEG. After 2-3 hours concentrated suspension was removed from dialysis tubing and centrifuged at 1600g for 5 min. The supernatant contained Fe@C-NH_2/BSA nanoparticles activated with glutaraldehyde was added to 10 mg/ml protein G solution in PBS under vortex stirring. Final protein G to Fe@C-NH_2/BSA ratio was 80 μg of protein G per 1 mg of nanoparticles. Conjugation of protein G was carried out overnight on rotating mixer at +4°C, glycine was added to 6 mM to quench unreacted aldehyde groups and the mixture was incubated at RT for one more hour. Unreacted protein G was removed by gel-chromatography (column XK 26/40, medium: Sepharose CL-6B, bed volume: 100 ml, elution speed: 0.65 ml/min, eluent: PBS). Fractions with the highest concentration of Fe@C-NH_2/BSA/Str nanoparticles were combined. Glycerol, BSA and glycine were added to the final concentrations of 20%, 1%, and 6 mM respectively. Conjugates were stored at +4°C. The concentration of nanoparticles in prepared conjugates were determined by absorbance at 450 nm.

Synthesis of casein-coated Fe@C-NH_2 functionalized with Streptococcal protein G (Fe@C-NH_2/Casein/G)

1278 μL of 8.8% casein in H_2O was mixed with 962 μL 0.1M acetic buffer pH 5 and 9.6 μL of 1M NaOH. After that, 2250 μL of 10 mg/ml Fe@C-NH_2 suspension was added dropwise under vortex stirring. Resulting suspension (4300 μL) was sonicated for 10 s and added dropwise under vortex stirring to an equal volume of 25% glutaraldehyde solution and incubated on rotating mixer at room temperature for 30 min. Then, 8.6 ml of nanoparticles activated with glutaraldehyde were passed through the chromatography column (column XK 26/40, medium: Sepharose CL-6B, bed volume: 160 ml, elution speed: 0.65 ml/min, eluent: PBS) to remove the excess of casein and glutaraldehyde. Fractions containing Fe@C-NH_2/Casein nanoparticles were collected and concentrated to approximately 3-4 ml. For concentration, the suspension was placed in dialysis tubing (10 kDa MWCO, 2 ml per cm) and covered with the layer of 35 kDa PEG. After 2-3 hours concentrated suspension was removed from dialysis tubing and centrifuged at 1600g for 5 min. The supernatant contained Fe@C-NH_2/Casein nanoparticles activated with glutaraldehyde was added

to 10 mg/ml protein G solution in PBS under vortex stirring. Final protein G to Fe@C-NH_2/Casein ratio was 80 μg of protein G per 1 mg of nanoparticles. Conjugation of protein G was carried out overnight on rotating mixer at +4°C, glycine was added to 6 mM to quench unreacted aldehyde groups, and the mixture was incubated at RT for one more hour. Unreacted protein G was removed by gel-chromatography (column XK 26/40, medium: Sepharose CL-6B, bed volume: 100 ml, elution speed: 0.65 ml/min, eluent: PBS). Fractions with the highest concentration of Fe@C-NH_2/BSA/Str nanoparticles were combined. Glycerol, BSA and glycine were added to the final concentrations of 20%, 1%, and 6 mM respectively. Conjugates were stored at +4°C. The concentration of nanoparticles in prepared conjugates was determined by absorbance at 450 nm.

Synthesis of Gelatin A/B-coated Fe@C-NH_2 functionalized with Streptococcal protein G (Fe@C-NH_2/Gelatin A/G and Fe@C-NH_2/Gelatin B/G)

1125 μL of 10% gelatin in H_2O was mixed with 1125 μL 0.1M acetic buffer pH 4. After that, 2250 μL of 10 mg/ml Fe@C-NH_2 suspension was added dropwise under vortex stirring; pH was adjusted to 7.25 by 1M NaOH. Resulting suspension (4300 μL) was sonicated for 5 min and added dropwise under vortex stirring to an equal volume of 25% glutaraldehyde solution and incubated on rotating mixer at +37°C for 30 min. All solutions were pre-warmed and kept at +37-40°C to prevent gelation. Then, 8.6 ml of nanoparticles activated with glutaraldehyde were passed through the chromatography column (column XK 26/40, medium: Sepharose CL-6B, bed volume: 160 ml, elution speed: 0.65 ml/min, eluent: PBS) to remove the excess of gelatin and glutaraldehyde. Fractions containing Fe@C-NH_2/Gelatin B nanoparticles were collected and concentrated to approximately 3-4 ml. For concentration, the suspension was placed in dialysis tubing (10 kDa MWCO, 2 ml per cm) and covered with the layer of 35 kDa PEG. After 2-3 hours concentrated suspension was removed from dialysis tubing and centrifuged at 1600g for 5 min. The supernatant contained Fe@C-NH_2/Gelatin B nanoparticles activated with glutaraldehyde was added to 10 mg/ml protein G solution in PBS under vortex stirring. Final protein G to Fe@C-NH_2/Gelatin B ratio was 80 μg of protein G per 1 mg of nanoparticles. Conjugation of protein G was carried out overnight on rotating mixer at +4°C, glycine was added to 6 mM to quench unreacted aldehyde groups, and the mixture was incubated at RT for one more hour. Unreacted protein G was removed by gel-chromatography (column XK 26/40, medium: Sepharose CL-6B, bed volume: 100 ml, elution speed: 0.65 ml/min, eluent: PBS). Fractions with the highest concentration of Fe@C-NH_2/BSA/Str nanoparticles were combined. Glycerol, BSA and glycine were added to the final concentrations of 20%, 1%, and 6 mM respectively. Conjugates were stored at +4°C. The concentration of nanoparticles in prepared conjugates was determined by absorbance at 450 nm.

Synthesis of BSA-coated Fe@C-NH_2 functionalized with streptavidin (Fe@C-NH_2/BSA/Str)

2250 μL of 10 mg/ml Fe@C-NH_2 suspension was added dropwise under vortex stirring to 625 μL of 20% BSA in H_2O, then 1595 μL of PBS and 30 μL of 1M NaOH were added to adjust pH to 7.2-7.6. Resulting suspension (4300 μL) was sonicated for 10 s and added dropwise under vortex stirring to an equal volume of 25% glutaraldehyde solution and incubated on rotating mixer at room temperature for 30 min. Then, 8.6 ml of nanoparticles activated with glutaraldehyde were passed through the chromatography column (column XK 26/40, medium: Sepharose CL-6B, bed volume: 160 ml, elution speed: 0.65 ml/min, eluent: PBS) to remove the excess of BSA and glutaraldehyde. Fractions containing Fe@C-NH_2/BSA nanoparticles were collected and concentrated to approximately 3-4 ml. For concentrations, the suspension was placed in dialysis tubing (10 kDa MWCO, 2 ml per cm) and covered with the layer of 35 kDa PEG. After 2-3 hours concentrated suspension was removed from dialysis tubing and centrifuged at 1600g for 5 min. The supernatant contained Fe@C-NH_2/BSA nanoparticles activated with glutaraldehyde was added to 10 mg/ml streptavidin solution in PBS under vortex stirring. Final streptavidin to Fe@C-NH_2/BSA ratio was 80 μg of streptavidin per 1 mg of nanoparticles. Conjugation of streptavidin was carried out overnight on rotating mixer at +4°C, glycine and BSA were added to 6 mM and 1 mg/ml respectively to quench unreacted aldehyde groups, and the mixture was incubated at RT for one more hour. Unreacted streptavidin was removed by gel-chromatography (column XK 26/40, medium: Sepharose CL-6B, bed volume: 100 ml, elution speed: 0.65 ml/min, eluent: PBS). Fractions with the highest concentration of Fe@C-NH_2/BSA/Str

nanoparticles were combined. Conjugates were stored at +4°C in PBS without any stabilizers. The concentration of nanoparticles in prepared conjugates were determined by absorbance at 450 nm.

Synthesis of casein-coated Fe@C-NH_2 functionalized with streptavidin (Fe@C-NH_2/Casein/Str)

1278 µL of 8.8% casein in H_2O was mixed with 962 µL 0.1M acetic buffer pH 5 and 9.6 µL of 1M NaOH. After that, 2250 µL of 10 mg/ml Fe@C-NH_2 suspension was added dropwise under vortex stirring. Resulting suspension (4300 µL) was sonicated for 10 s and added dropwise under vortex stirring to an equal volume of 25% glutaraldehyde solution and incubated on rotating mixer at room temperature for 30 min. Then, 8.6 ml of nanoparticles activated with glutaraldehyde were passed through the chromatography column (column XK 26/40, medium: Sepharose CL-6B, bed volume: 160 ml, elution speed: 0.65 ml/min, eluent: PBS) to remove the excess of casein and glutaraldehyde. Fractions containing Fe@C-NH_2/Casein nanoparticles were collected and concentrated to approximately 3-4 ml. For concentrations, the suspension was placed in dialysis tubing (10 kDa MWCO, 2 ml per cm) and covered with the layer of 35 kDa PEG. After 2-3 hours concentrated suspension was removed from dialysis tubing and centrifuged at 1600g for 5 min. The supernatant contained Fe@C-NH_2/Casein nanoparticles activated with glutaraldehyde was added to 10 mg/ml streptavidin solution in PBS under vortex stirring. Final streptavidin to Fe@C-NH_2/Casein ratio was 80 µg of streptavidin per 1 mg of nanoparticles. Conjugation of streptavidin was carried out overnight on rotating mixer at +4°C, glycine was added to 6 mM to quench unreacted aldehyde groups, and the mixture was incubated at RT for one more hour. Unreacted streptavidin was removed by gel-chromatography (column XK 26/40, medium: Sepharose CL-6B, bed volume: 100 ml, elution speed: 0.65 ml/min, eluent: PBS). Fractions with the highest concentration of Fe@C-NH_2/Casein/Str nanoparticles were combined. Conjugates were stored at +4°C in PBS without any stabilizers. The concentration of nanoparticles in prepared conjugates was determined by absorbance at 450 nm.

Synthesis of Gelatin A/B-coated Fe@C-NH_2 functionalized with streptavidin (Fe@C-NH_2/Gelatin A/Str and Fe@C-NH_2/Gelatin B/Str)

1125 µL of 10% gelatin in H_2O was mixed with 1125 µL 0.1M acetic buffer pH 4. After that, 2250 µL of 10 mg/ml Fe@C-NH_2 suspension was added dropwise under vortex stirring; pH was adjusted to 7.25 by 1M NaOH. Resulting suspension (4300 µL) was sonicated for 5 min and added dropwise under vortex stirring to an equal volume of 25% glutaraldehyde solution and incubated on rotating mixer at +37°C for 30 min. All solutions were pre-warmed and kept at +37-40°C to prevent gelation. Then, 8.6 ml of nanoparticles activated with glutaraldehyde were passed through the chromatography column (column XK 26/40, medium: Sepharose CL-6B, bed volume: 160 ml, elution speed: 0.65 ml/min, eluent: PBS) to remove the excess of gelatin and glutaraldehyde. Fractions containing Fe@C-NH_2/Gelatin B nanoparticles were collected and concentrated to approximately 3-4 ml. For concentrations, the suspension was placed in dialysis tubing (10 kDa MWCO, 2 ml per cm) and covered with the layer of 35 kDa PEG. After 2-3 hours concentrated suspension was removed from dialysis tubing and centrifuged at 1600g for 5 min. The supernatant contained Fe@C-NH_2/Gelatin B nanoparticles activated with glutaraldehyde was added to 10 mg/ml streptavidin solution in PBS under vortex stirring. Final streptavidin to Fe@C-NH_2/Gelatin B ratio was 80 µg of streptavidin per 1 mg of nanoparticles. Conjugation of streptavidin was carried out overnight on rotating mixer at +4°C, glycine was added to 6 mM to quench unreacted aldehyde groups and the mixture was incubated at RT for one more hour. Unreacted streptavidin was removed by gel-chromatography (column XK 26/40, medium: Sepharose CL-6B, bed volume: 100 ml, elution speed: 0.65 ml/min, eluent: PBS). Fractions with the highest concentration of Fe@C-NH_2/BSA/Str nanoparticles were combined. Conjugates were stored at +4°C in PBS without any stabilizers. The concentration of nanoparticles in prepared conjugates were determined by absorbance at 450 nm.

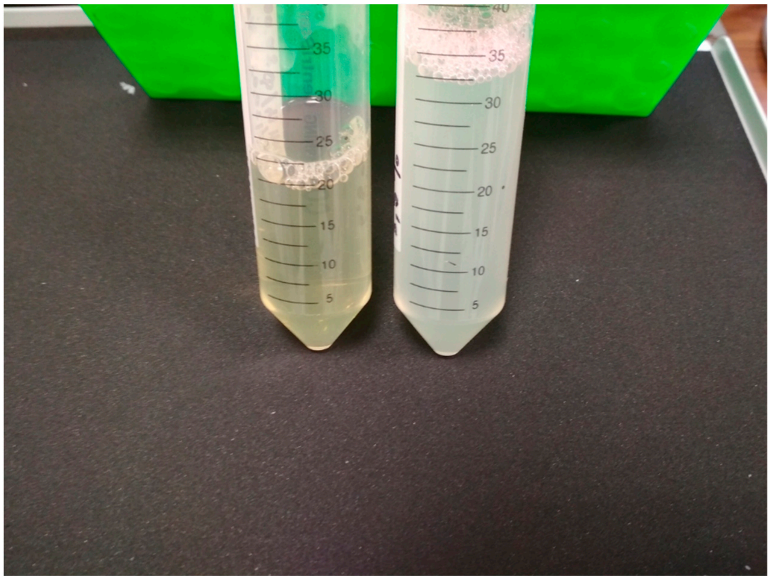

Figure 1. Casein solution before (right) and after (left) removal of micelles.

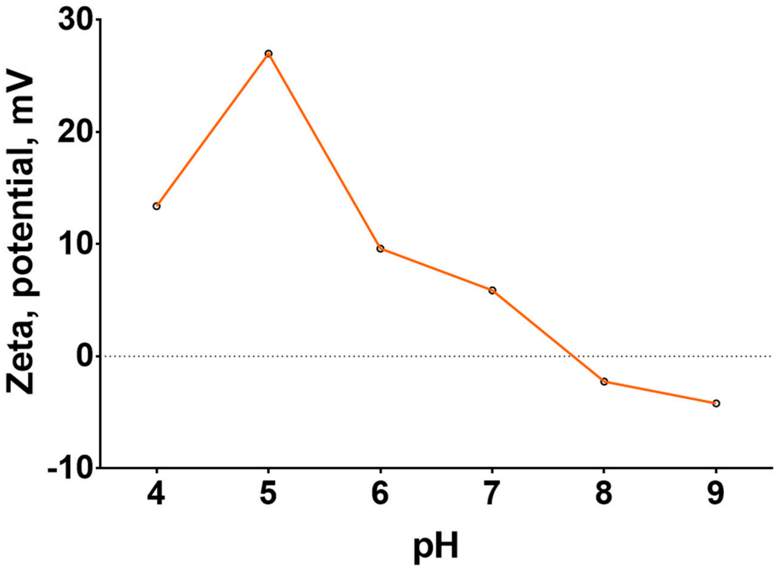

Figure 2. Zeta potential of Fe@C-NH_2.

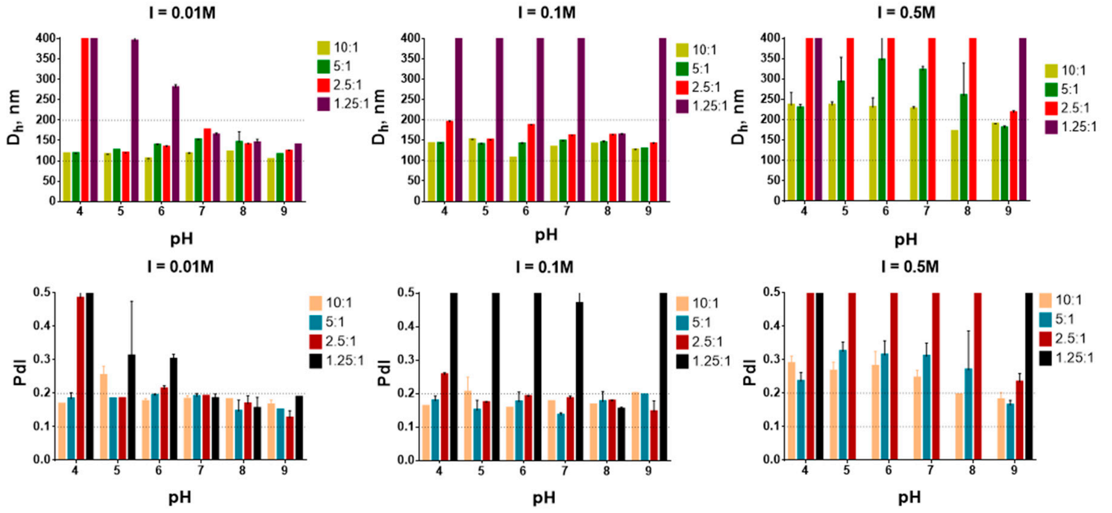

Figure S3. Influence of pH, ionic strength and protein-to-nanoparticle mass ratio on the size (hydrodynamic diameter) and polydispersity index of BSA-coated Fe@C-NH_2 .

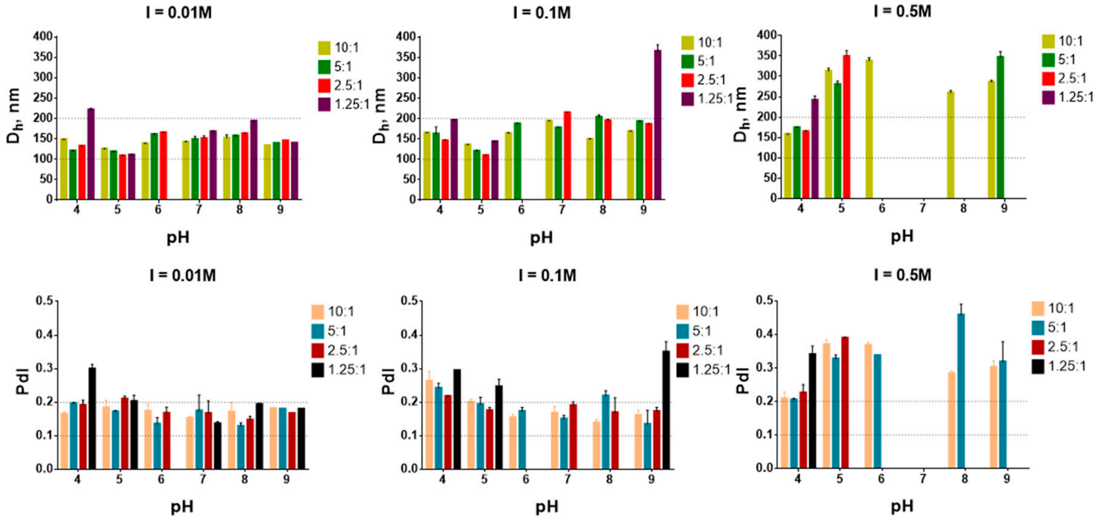

Figure S4. Influence of pH, ionic strength and protein-to-nanoparticle mass ratio on the size (hydrodynamic diameter) and polydispersity index of casein-coated Fe@C-NH_2. The absence of bars reveals a severe aggregation of nanoclusters in the corresponding samples.

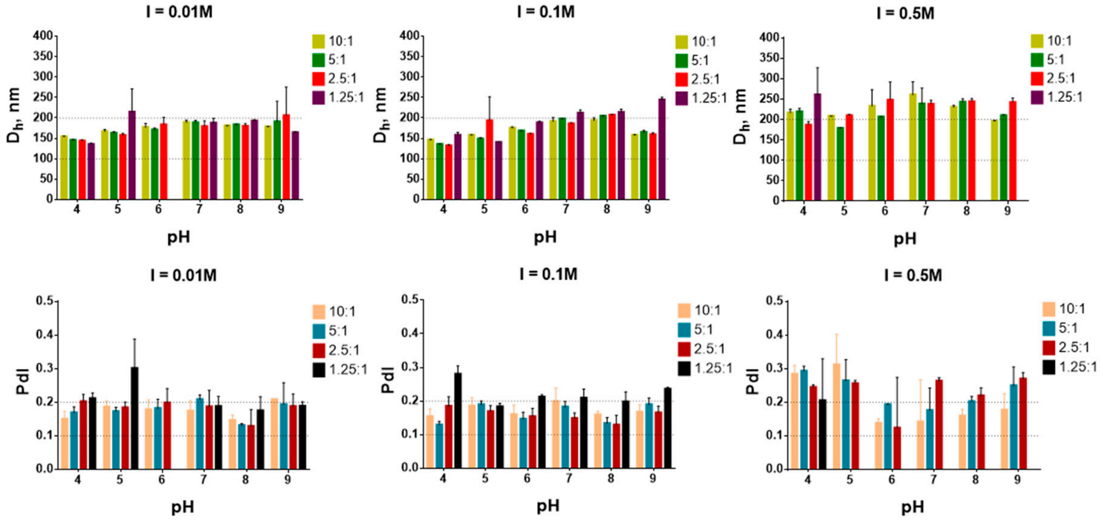

Figure 5. Influence of pH, ionic strength and protein-to-nanoparticle mass ratio on the size (hydrodynamic diameter) and polydispersity index of gelatin B-coated Fe@C-NH_2. The absence of bars reveals a severe aggregation of nanoclusters in the corresponding samples.

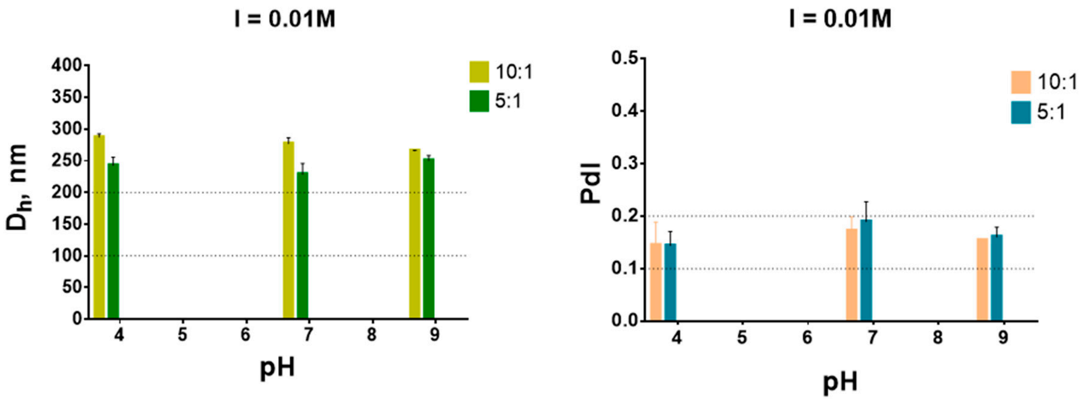

Figure 6. Influence of pH and protein-to-nanoparticle mass ratio on the size (hydrodynamic diameter) and polydispersity index of Gelatin A-coated Fe@C-NH_2. The absence of bars reveals a severe aggregation of nanoclusters in the corresponding samples.

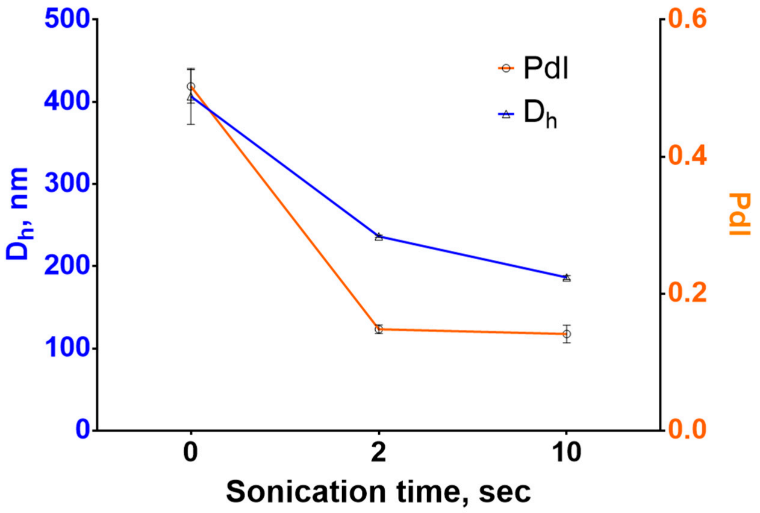

Figure 7. Influence of sonication duration on size and polydispersity of Gelatin B-stabilized Fe@C-NH_2 (pH 8, I=0.5).

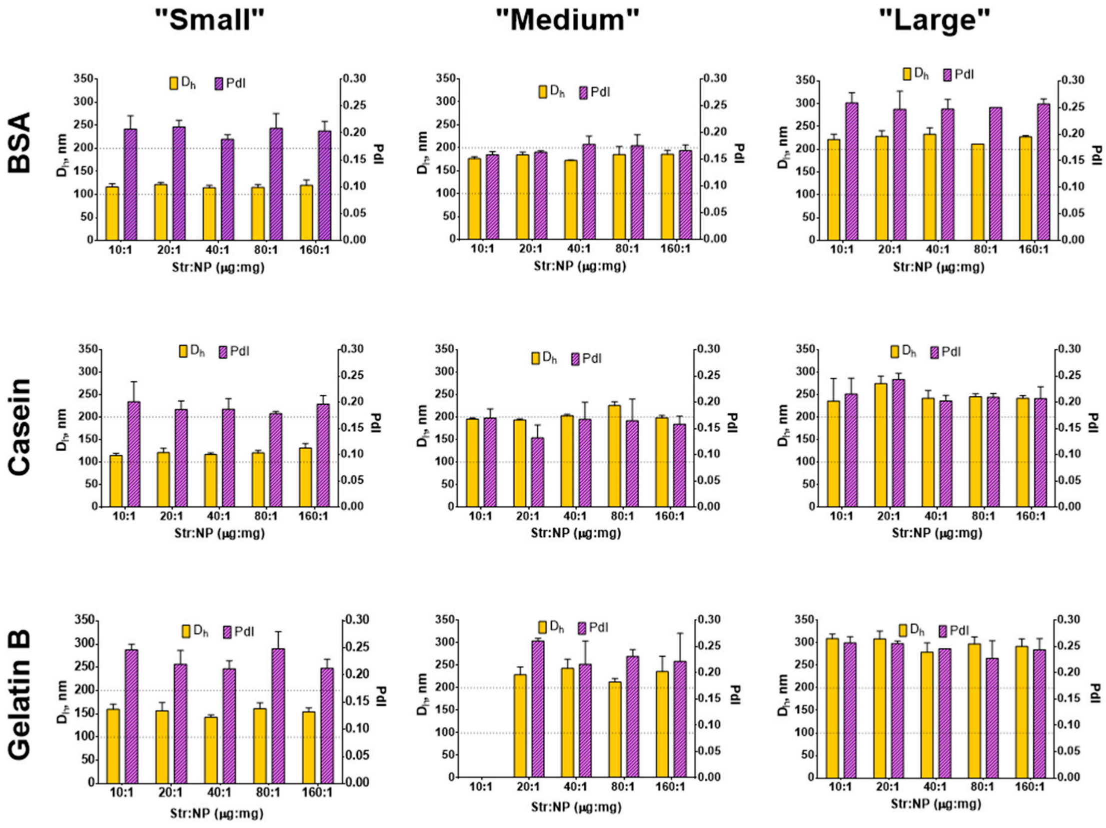

Figure 8. Hydrodynamic diameter and polydispersity of "small", "medium" and "large" Fe@C-NH_2/Protein/Str. Streptavidin to nanoparticles ratio (µg:mg) on the x axis. The absence of bars reveals a severe aggregation of nanoclusters in the corresponding samples.

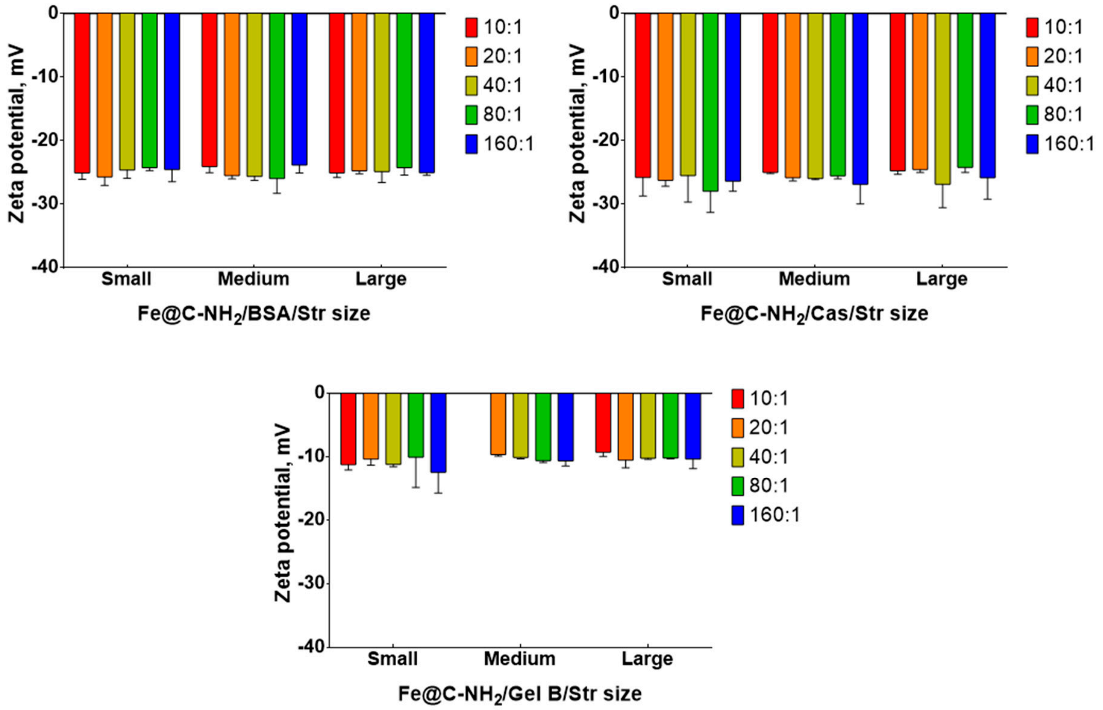

Figure S9. Zeta potential of “small”, “medium” and “large” Fe@C-NH_2/Protein/Str. Conjugates were prepared using various streptavidin to nanoparticles ratio, μg:mg (see legend). The absence of bars reveals a severe aggregation of nanoclusters in the corresponding samples.

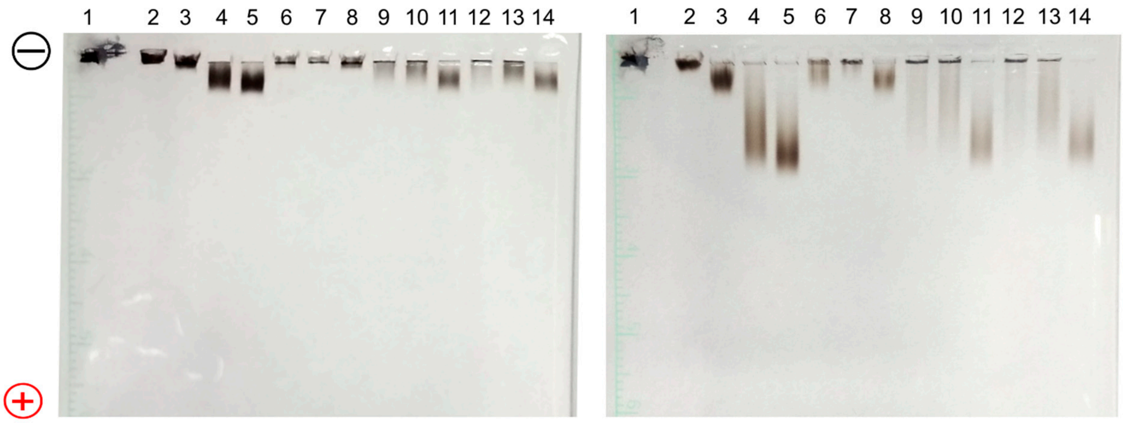

Figure S10. Migration of Fe@C-NH_2/protein/Str and Fe@C-NH_2/protein/G in 0.5% agarose gel (Tris-HCl pH 8, 75V). Photos were taken 5 (left) and 15 (right) minutes after start. Lanes: 1 - Fe@C-NH_2; 2 - Fe@C-NH_2/Gelatin A/G; 3 - Fe@C-NH_2/Gelatin B/G; 4 - Fe@C-NH_2/Casein/G; 5 - Fe@C-NH_2/BSA/G; 6 - Fe@C-NH_2/Gelatin B/Str “Medium”; 7 - Fe@C-NH_2/Gelatin B/Str “Large”; 8 - Fe@C-NH_2/Gelatin B/Str “Small”; 9 - Fe@C-NH_2/Casein/Str “Large”; 10 - Fe@C-NH_2/Casein/Str “Medium”; 11 - Fe@C-NH_2/Casein/Str “Small”; 12 - Fe@C-NH_2/BSA/Str “Large”; 13 - Fe@C-NH_2/BSA/Str “Medium”; 14 - Fe@C-NH_2/BSA/Str “Small”.

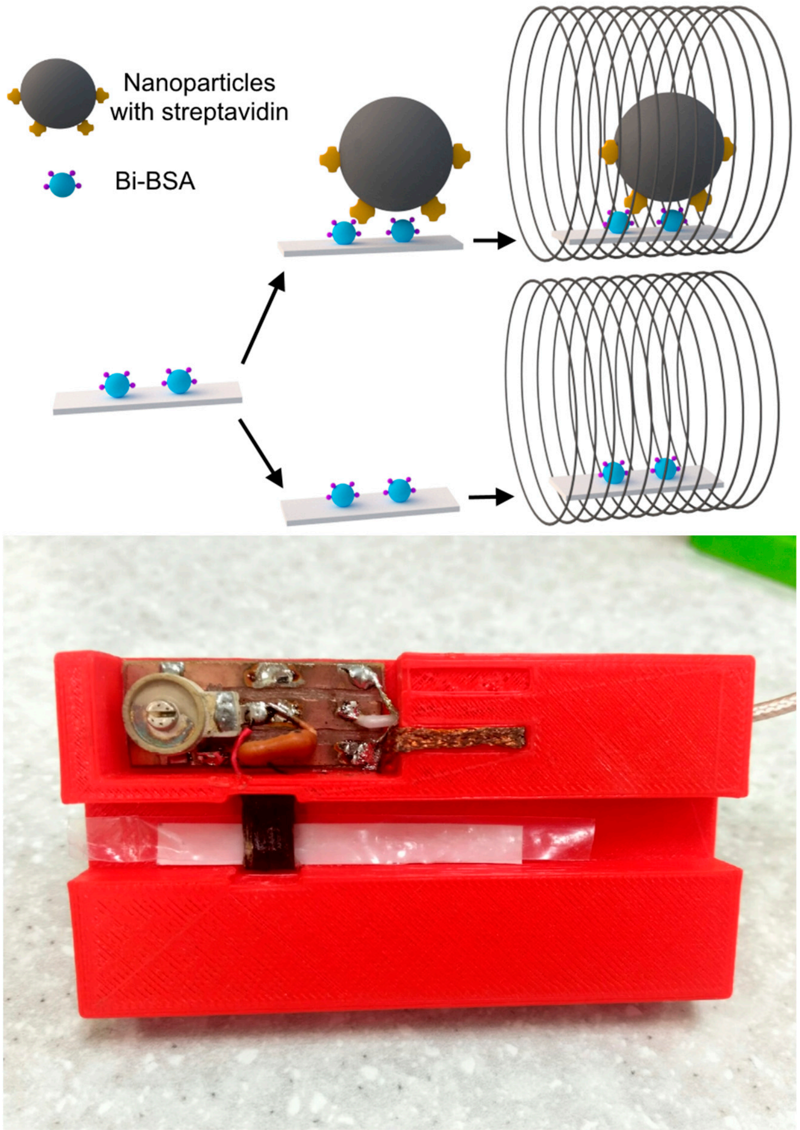

Figure 11. Principle of NMR-assay of Bi-BSA on nitrocellulose membrane (top). Sample holder with test strip (bottom).

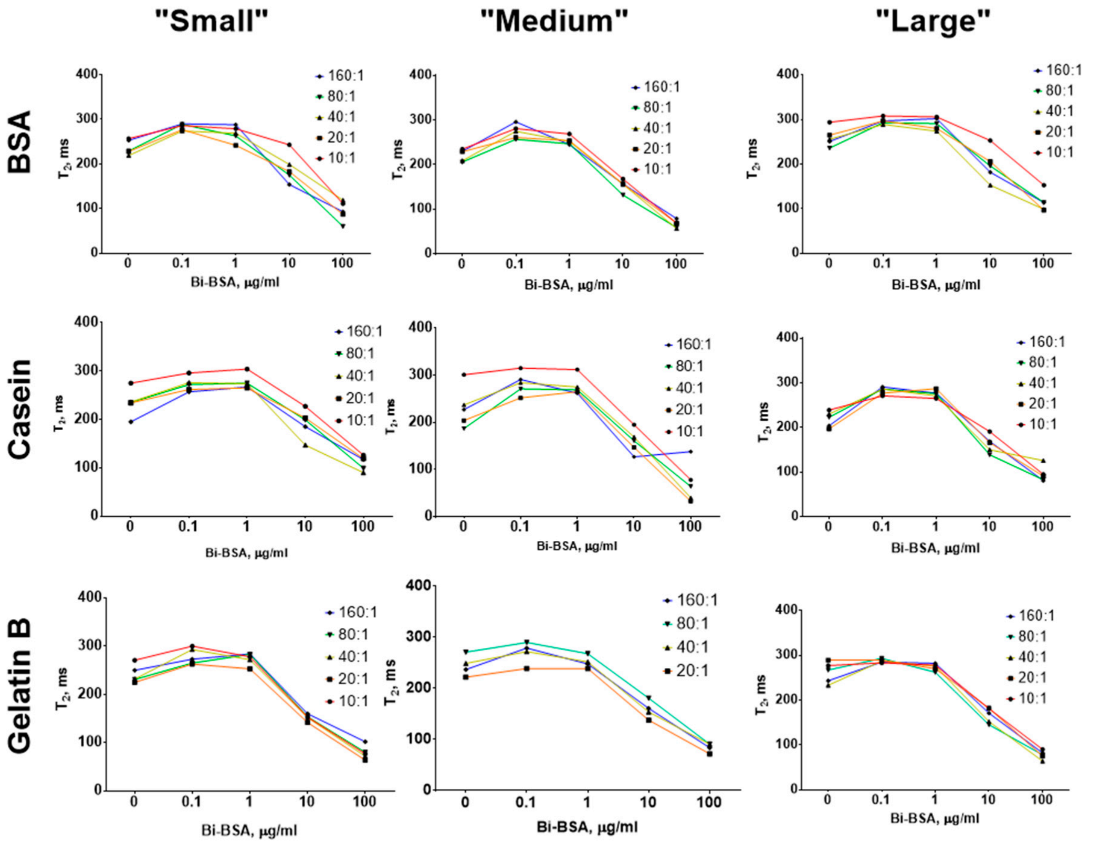

Figure 12. Functional activity of "small", "medium" and "large" Fe@C-NH_2/Protein/Str in solid phase NMR-assay. Conjugates were prepared using various streptavidin to nanoparticles ratio (µg:mg): 10:1 (red), 20:1 (orange), 40:1 (yellow), 80:1 (green), 160:1 (blue). Bi-BSA - biotinylated BSA.

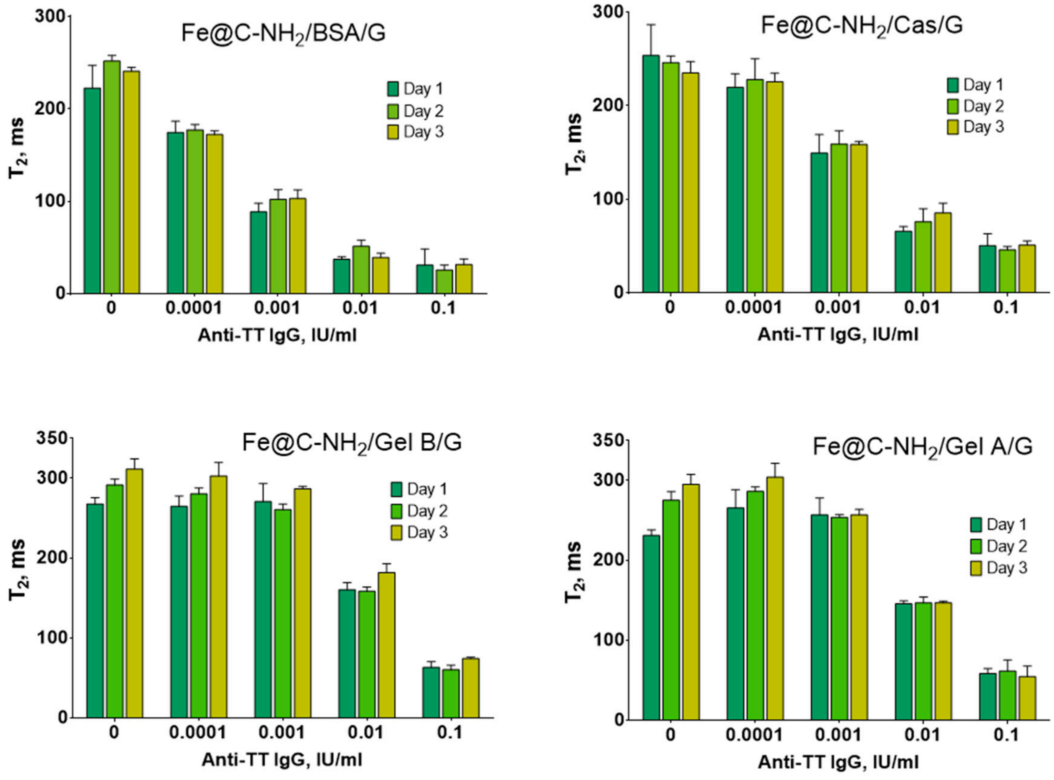

Figure 13. Day-to-day reproducibility of anti-tetanus toxoid NMR-assay. Three independent assays were performed in different days for each conjugate.

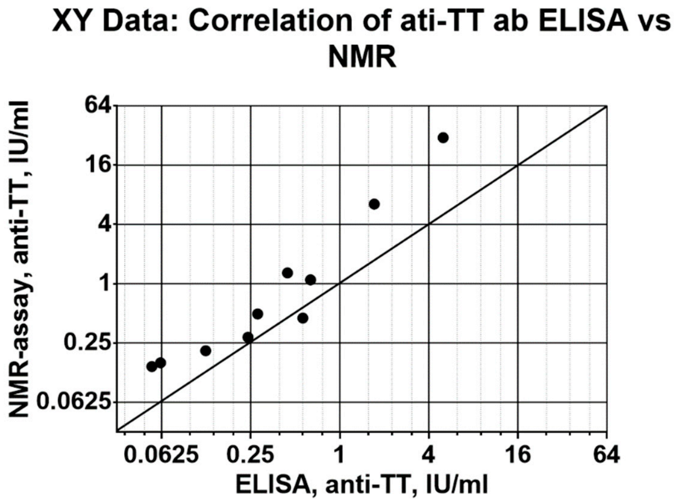

Figure 14. Comparison of ELISA and NMR-assay of IgG against tetanus toxoid.

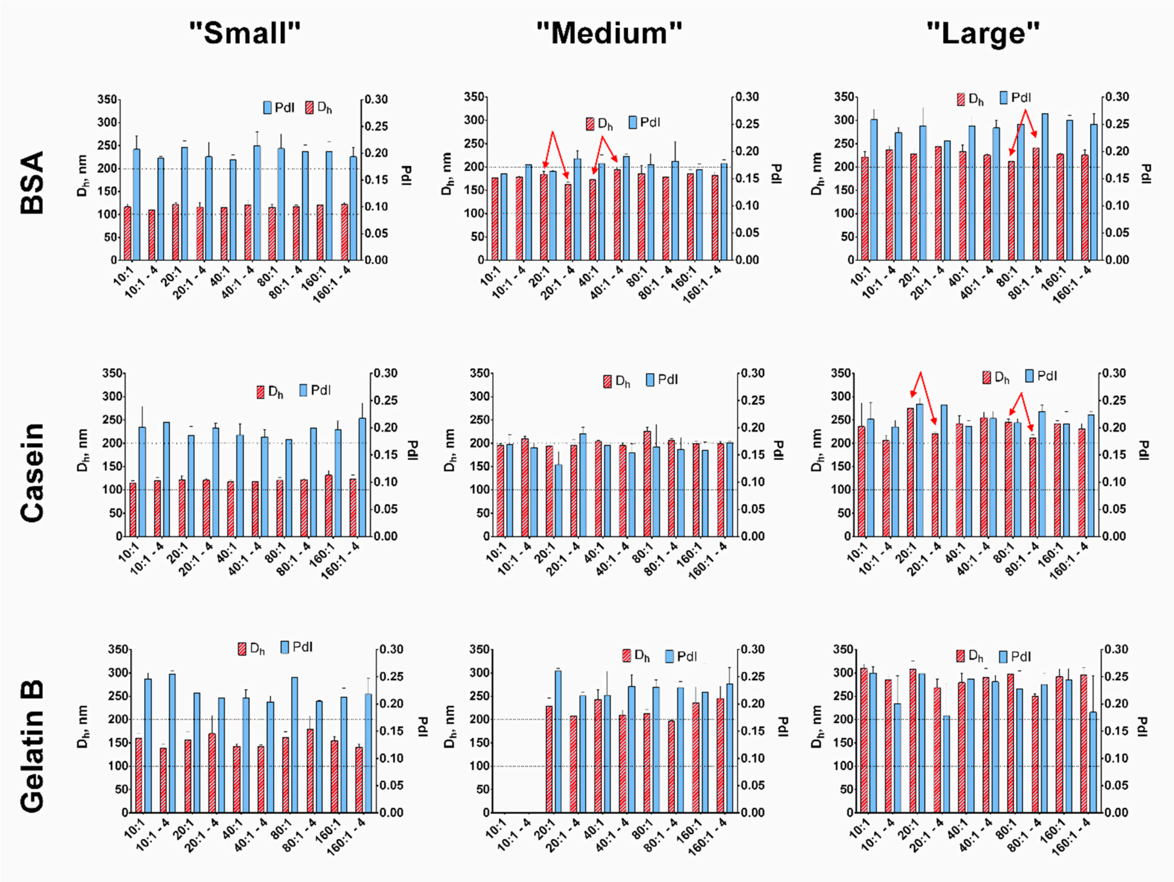

Figure 15. Storage stability of Fe@C-NH_2/Protein/Str nanoparticles, obtained in the course of size tuning experiments. Streptavidin to nanoparticles ratio (µg:mg) on the x axis. Digit 4 indicates conjugates stored for 4 months. Arrows depict samples with altered size (both increase and decrease).

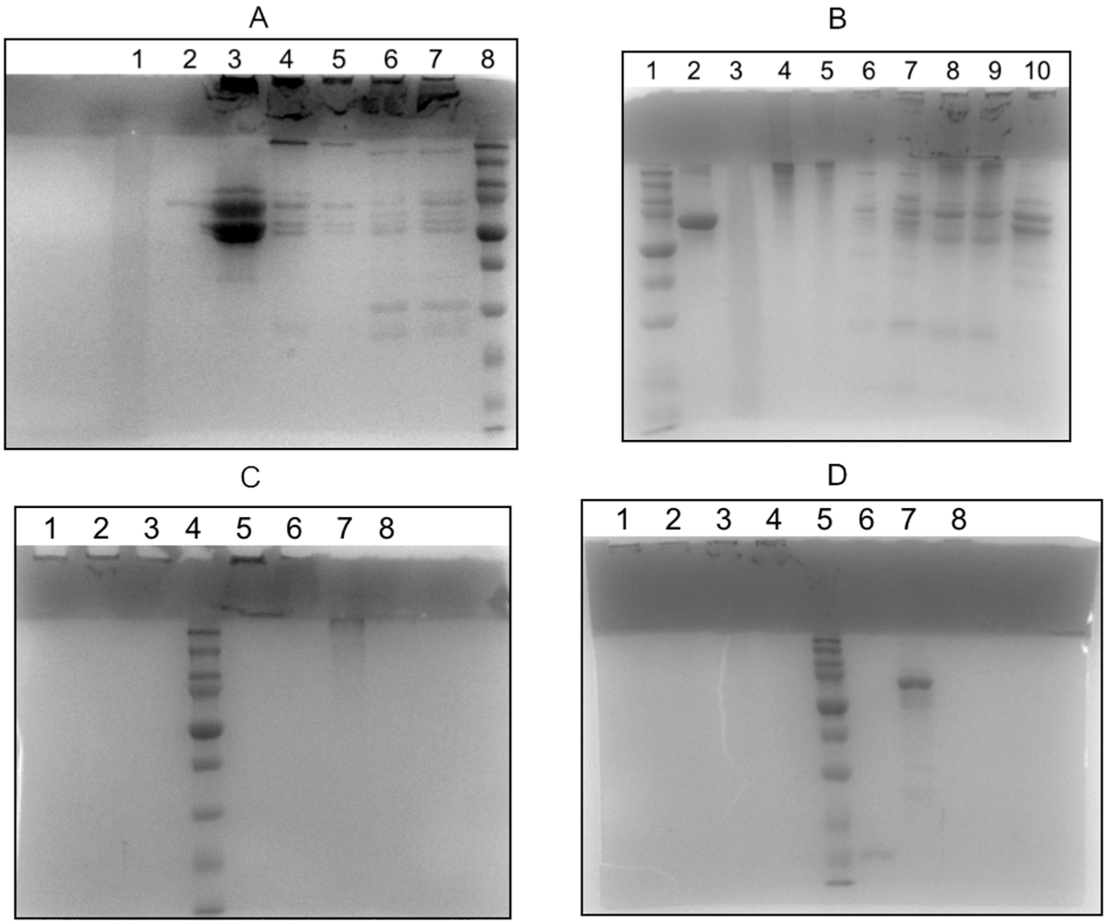

Figure 16. Protein corona of Fe@C-NH_2/Protein/Str after incubation in blood serum and plasma. **A**: lane 1 - casein, lane 2 - plasma, lane 3 - Fe@C-NH_2 (1 hour in plasma), lane 4 - Fe@C-NH_2/Gelatin B/Str (1 hour in plasma), lane 5 - Fe@C-NH_2/Gelatin A/Str (1 hour in plasma), lane 6 - Fe@C-NH_2/Casein/Str (1 hour in plasma), lane 7 - Fe@C-NH_2/BSA/Str (1 hour in plasma), lane 8 - protein markers. **B**: lane 1 - protein marker, lane 2 - BSA, lane 3 - casein, lane 4 - gelatin A, lane 5 - gelatin B, lane 6 - Fe@C-NH_2/BSA/Str (1 hour in serum), lane 7 - Fe@C-NH_2/Casein/Str (1 hour in serum), lane 8 - Fe@C-NH_2/Gelatin A/Str (1 hour in serum), lane 9 - Fe@C-NH_2/Gelatin B/Str (1 hour in serum), lane 10 - Fe@C-NH_2 (1 hour in serum). **C**: lane 1 - Fe@C-NH_2 (1 hour in PBS), lane 2 - Fe@C-NH_2/BSA/Str (1 hour in PBS), lane 3 - Fe@C-NH_2/Casein/Str (1 hour in PBS), lane 4 - protein markers, lane 5 - Fe@C-NH_2/Gelatin A/Str (1 hour in PBS), lane 6 - Fe@C-NH_2/Gelatin B/Str (1 hour in PBS), lane 7 - gelatin A, lane 8 - gelatin B. **D**: lane 1 - Fe@C-NH_2/BSA/Str (1 hour in PBS), lane 2 - Fe@C-NH_2/Casein/Str (1 hour in PBS), lane 3 - Fe@C-NH_2/Gelatin A/Str (1 hour in PBS), lane 4 - Fe@C-NH_2/Gelatin B/Str (1 hour in PBS), lane 5 - protein markers, lane 6 - streptavidin, lane 7 - blood serum, lane 8 - Fe@C-NH_2 (1 hour in PBS).

nanomaterials

Table 1. Properties of conjugates used in this study.

Group	Str(protein G):nanoparticles ratio, μg:mg	Coating	Attached molecule	D_h, nm	PdI	Relaxivity, $1/mM^{-1} \times s^{-1}$	Conc., mg/ml	Parent aminated nanoparticles	Were used in following experiments
Small	10:1	BSA	Streptavidin	116	0,207	265,3813	0,3888	AM-SIZE	Size tuning, long-term storage
Small	20:1	BSA	Streptavidin	121	0,211	241,8847	0,4779	AM-SIZE	Size tuning, long-term storage
Small	40:1	BSA	Streptavidin	114	0,188	236,8949	0,5103	AM-SIZE	Size tuning, long-term storage
Small	80:1	BSA	Streptavidin	115	0,208	234,6137	0,5022	AM-SIZE	Size tuning, long-term storage
Small	160:1	BSA	Streptavidin	120	0,203	236,5807	0,5427	AM-SIZE	Size tuning, long-term storage
Medium	10:1	BSA	Streptavidin	176	0,158	262,0929	0,4536	AM-SIZE	Size tuning, long-term storage
Medium	20:1	BSA	Streptavidin	184	0,163	284,4745	0,4293	AM-SIZE	Size tuning, long-term storage
Medium	40:1	BSA	Streptavidin	172	0,178	322,8937	0,4212	AM-SIZE	Size tuning, long-term storage
Medium	80:1	BSA	Streptavidin	185	0,175	272,6557	0,4941	AM-SIZE	Size tuning, long-term storage
Medium	160:1	BSA	Streptavidin	186	0,166	300,4936	0,5022	AM-SIZE	Size tuning, long-term storage
Large	10:1	BSA	Streptavidin	221	0,258	186,0687	0,4131	AM-SIZE	Size tuning, long-term storage
Large	20:1	BSA	Streptavidin	228	0,246	230,058	0,3726	AM-SIZE	Size tuning, long-term storage
Large	40:1	BSA	Streptavidin	233	0,247	214,7827	0,3969	AM-SIZE	Size tuning, long-term storage
Large	80:1	BSA	Streptavidin	211	0,25	223,3444	0,3645	AM-SIZE	Size tuning, long-term storage
Large	160:1	BSA	Streptavidin	227	0,257	219,0282	0,3564	AM-SIZE	Size tuning, long-term storage
Small	10:1	Casein	Streptavidin	114	0,201	310,8042	0,3483	AM-SIZE	Size tuning, long-term storage
Small	20:1	Casein	Streptavidin	121	0,186	345,4113	0,3564	AM-SIZE	Size tuning, long-term storage
Small	40:1	Casein	Streptavidin	117	0,186	327,2012	0,3807	AM-SIZE	Size tuning, long-term storage
Small	80:1	Casein	Streptavidin	120	0,178	344,5608	0,3483	AM-SIZE	Size tuning, long-term storage
Small	160:1	Casein	Streptavidin	131	0,196	313,6018	0,3807	AM-SIZE	Size tuning, long-term storage
Medium	10:1	Casein	Streptavidin	195	0,169	340,1031	0,3726	AM-SIZE	Size tuning, long-term storage
Medium	20:1	Casein	Streptavidin	194	0,132	335,4058	0,3483	AM-SIZE	Size tuning, long-term storage
Medium	40:1	Casein	Streptavidin	203	0,167	318,391	0,405	AM-SIZE	Size tuning, long-term storage
Medium	80:1	Casein	Streptavidin	225	0,164	337,1553	0,3726	AM-SIZE	Size tuning, long-term storage
Medium	160:1	Casein	Streptavidin	198	0,158	321,38	0,2997	AM-SIZE	Size tuning, long-term storage
Large	10:1	Casein	Streptavidin	235	0,215	345,0889	0,1863	AM-SIZE	Size tuning, long-term storage
Large	20:1	Casein	Streptavidin	274	0,243	330,2673	0,3078	AM-SIZE	Size tuning, long-term storage
Large	40:1	Casein	Streptavidin	242	0,202	303,6793	0,3402	AM-SIZE	Size tuning, long-term storage
Large	80:1	Casein	Streptavidin	245	0,209	327,5499	0,3483	AM-SIZE	Size tuning, long-term storage
Large	160:1	Casein	Streptavidin	242	0,207	309,1843	0,3402	AM-SIZE	Size tuning, long-term storage
Small	10:1	Gelatin B	Streptavidin	159	0,246	266,7957	0,26125	AM-SIZE	Size tuning, long-term storage
Small	20:1	Gelatin B	Streptavidin	156	0,22	276,309	0,3325	AM-SIZE	Size tuning, long-term storage
Small	40:1	Gelatin B	Streptavidin	142	0,211	292,5403	0,30875	AM-SIZE	Size tuning, long-term storage
Small	80:1	Gelatin B	Streptavidin	160	0,248	298,0891	0,324583333	AM-SIZE	Size tuning, long-term storage
Small	160:1	Gelatin B	Streptavidin	154	0,212	306,3228	0,3325	AM-SIZE	Size tuning, long-term storage
Medium	10:1	Gelatin B	Streptavidin	aggregated	aggregated	aggregated	aggregated	AM-SIZE	Size tuning, long-term storage
Medium	20:1	Gelatin B	Streptavidin	229	0,26	377,1929	0,2997	AM-SIZE	Size tuning, long-term storage
Medium	40:1	Gelatin B	Streptavidin	243	0,216	324,2472	0,2592	AM-SIZE	Size tuning, long-term storage
Medium	80:1	Gelatin B	Streptavidin	212	0,231	296,7454	0,2754	AM-SIZE	Size tuning, long-term storage
Medium	160:1	Gelatin B	Streptavidin	236	0,221	312,0377	0,2673	AM-SIZE	Size tuning, long-term storage
Large	10:1	Gelatin B	Streptavidin	309	0,257	308,3217	0,2025	AM-SIZE	Size tuning, long-term storage
Large	20:1	Gelatin B	Streptavidin	308	0,255	282,6507	0,2106	AM-SIZE	Size tuning, long-term storage
Large	40:1	Gelatin B	Streptavidin	279	0,246	301,3985	0,2025	AM-SIZE	Size tuning, long-term storage
Large	80:1	Gelatin B	Streptavidin	297	0,227	308,0283	0,1944	AM-SIZE	Size tuning, long-term storage
Large	160:1	Gelatin B	Streptavidin	292	0,244	314,5411	0,1782	AM-SIZE	Size tuning, long-term storage

	80:1	BSA	Protein G	105	0,163	335,7127	1,0908	AM-G	Long-term storage, IgG NMR-assay, stability in complex media, colloidal stability, thermal stability
	80:1	Casein	Protein G	132	0,161	377,0426	0,8964	AM-G	Long-term storage, IgG NMR-assay, stability in complex media, colloidal stability, thermal stability
	80:1	Gelatin B	Protein G	153	0,186	368,492	0,7668	AM-G	Long-term storage, IgG NMR-assay, stability in complex media, colloidal stability, thermal stability
	80:1	Gelatin A	Protein G	167	0,18	389,485	0,5724	AM-G	Long-term storage, IgG NMR-assay, stability in complex media, colloidal stability, thermal stability
	40:1	BSA	Streptavidin	111	0,161	204,8375	1,586	AM-Str	Protein corona, proteolysis, stability in complex media
	40:1	Casein	Streptavidin	119	0,154	221,7823	1,22	AM-Str	Protein corona, proteolysis, stability in complex media
	40:1	Gelatin B	Streptavidin	154	0,131	232,5224	0,71291666 7	AM-Str	Protein corona, proteolysis, stability in complex media
	40:1	Gelatin A	Streptavidin	186	0,181	232,1508	0,73708333 3	AM-Str	Protein corona, proteolysis, stability in complex media
AM-SIZE	none	NH2	none	96,42	0,248	285,8172	8,1	none	
AM-G	none	NH2	none	110,45	0,251	335,5421	10,8	none	
AM-Str	none	NH2	none	109,2	0,272	250,7953	12,2	none	

© 2019 by the authors. Submitted for possible open access publication under the terms and conditions of the Creative Commons Attribution (CC BY) license (http://creativecommons.org/licenses/by/4.0/).
